# Supplementary material for: Food Consumption in Adolescents and Young Adults: Age-Specific Socio-Economic and Cultural Disparities (Belgian Food Consumption Survey 2014)
Source: Nutrients. 2019 Jul 4;11(7):1520. doi: 10.3390/nu11071520 (PMC6683246; doi:10.3390/nu11071520)
Supplement: Supplementary file 1 [file nutrients-11-01520-s001.pdf]

Supplementary Materials: Food Consumption in Adolescents and Young Adults: Age-Specific Socio-Economic and Cultural Disparities (Belgian Food Consumption Survey 2014)

Lucille Desbouys, Karin De Ridder, Manon Rouche and Katia Castetbon

Table S1. Adjusted for total energy intake mean consumption (g/day) of four food groups according to socio-economic and cultural characteristics and age group. Belgian Food Consumption Survey 2014.

	Age category							
	10-13 years		14-17 years		18-25 years		26-39 years	
	**b (ES)[a]**	***p***	**b (ES)[a]**	***p***	**b (ES)[a]**	***p***	**b (ES)[a]**	***p***
**Fruits and vegetables**								
**Gender**		0.72		**<0.01**		0.09		0.31
Male	-5.0 (13.8)		**-53.0 (17.4)**		-49.3 (29.4)		-25.1 (24.5)	
Female	0		0		0		0	
**Household type**		0.27		**0.03**				
Two-parent family	0		0					
Single-parent family	-15.2 (13.7)		**-34.1 (16.0)**		-		-	
Single					-70.0 (56.0)	**0.02**	-80.0 (47.1)	**0.01**
Single-parent family					**-130.1 (37.9)**		**-105.7 (43.3)**	
Couple without children	-		-		0		0	
Two-parent family					**-68.5 (31.3)**		-30.4 (38.8)	
Other					-79.6 (42.2)		15.2 (60.4)	
**Highest education level in the**		**<0.001**		**<0.01**		**0.02**		
**household**	**-71.6 (15.9)**		**-52.5 (16.8)**		**-74.0 (29.6)**			
Secondary education or lower	**-55.8 (17.6)**		-6.4 (20.2)		-20.2 (30.5)		-	
Bachelor's degree or equivalent	0		0		0			
Postgraduate education								
**Education level of the responder**								0.07
Secondary education or lower							**-64.1 (29.2)**	
Bachelor's degree or equivalent	-		-		-		-21.6 (28.7)	
Postgraduate education							0	
**Maternal occupation**		**<0.01**		**<0.001**				
Inactive	-51.3 (27.7)		-55.7 (51.8)					
Manual worker	**-68.2 (29.6)**		**-117.5 (52.5)**					
Self-employed	-18.0 (29.5)		-35.8 (53.3)		-		-	
Employee or intermediate	-13.0 (25.6)		-35.4 (51.9)					
occupation	0		0					
Managerial or academic	**-70.6 (28.8)**		-45.9 (57.0)					
Not applicable (no mother declared)								
**Working status of the responder**						0.23		
Student					-18.2 (25.2)			
Inactive	-		-		-57.3 (33.3)		-	

Active					0			
**Occupation of the responder**								0.07
Inactive							-54.5 (35.7)	
Manual worker							-57.7 (41.7)	
Self-employed	-		-		-		-14.2 (47.4)	
Employee or intermediate							11.3 (34.9)	
occupation							0	
Managerial or academic								
**Country of birth**		0.78		**<0.001**		0.59		0.40
Belgium	0		0		0		0	
EU	-14.6 (20.8)		**56.0 (23.4)**		17.4 (38.0)		3.5 (37.5)	
Outside the EU	-0.3 (24.4)		**142.3 (41.2)**		74.6 (78.0)		66.2 (49.0)	
**Language spoken at home**		0.75		0.19		0.37		0.47
French and/or Dutch	0		0		0		0	
Mixed including French or Dutch	1.4 (16.1)		19.8 (23.6)		97.8 (70.6)		-45.5 (57.8)	
Language other than French or	-26.4 (35.5)		85.7 (51.4)		24.2 (82.0)		-37.1 (38.0)	
Dutch								
**Region of residency**		**<0.001**		<0.20		0.05		0.13
Flanders	0		0		0		0	
Brussels	-21.5 (21.1)		-23.6 (20.7)		-13.8 (58.8)		-28.7 (59.3)	
Wallonia	**-64.3 (13.5)**		-28.2 (16.8)		**-57.9 (23.8)**		**-43.5 (21.4)**	

Whole grain bread and cereals

**Gender**		0.14		0.07		0.91		0.13
Male	9.2 (6.3)		11.6 (6.5)		-1.2 (9.8)		14.7 (9.6)	
Female	0		0		0		0	
**Household type**		**0.02**		**0.03**				
Two-parent family	0		0					
Single-parent family	**-13.4 (5.6)**		**-13.9 (6.3)**		-		-	
Single					-4.7 (38.4)	0.09	-14.5 (15.3)	0.11
Single-parent family					-51.1 (26.2)		**-30.7 (15.0)**	
Couple without children	-		-		0		0	
Two-parent family					-30.8 (25.6)		-2.2 (13.7)	
Other					-41.5 (26.2)		-3.8 (22.2)	
**Highest education level in the**		**0.01**		**<0.01**		0.13		
**household**	**-23.7 (8.0)**		**-23.3 (6.8)**		-22.8 (12.7)			
Secondary education or lower	**-15.9 (8.0)**		-11.2 (8.5)		-6.1 (13.3)		-	
Bachelor's degree or equivalent	0		0		0			
Postgraduate education								
**Education level of the responder**								0.42
Secondary education or lower							-15.5 (11.8)	
Bachelor's degree or equivalent	-		-		-		-5.4 (11.2)	
Postgraduate education							0	
**Maternal occupation**		**0.01**		0.09				
Inactive	-20.4 (17.7)		13.6 (10.1)					

Manual worker	**-39.6 (17.1)**		15.7 (10.5)					
Self-employed	0		21.4 (12.8)		-		-	
Employee or intermediate	-17.4 (16.7)		**25.7 (9.4)**					
occupation	-8.4 (27.1)		0					
Managerial or academic	-19.0 (18.4)		**28.3 (14.2)**					
Not applicable (no mother declared)								
**Working status of the responder**						0.29		
Student					-18.0 (11.2)			
Inactive	-		-		-13.2 (20.8)		-	
Active					0			
**Occupation of the responder**								0.13
Inactive							**-28.0 (11.6)**	
Manual worker							-7.2 (15.3)	
Self-employed	-		-		-		-25.5 (16.8)	
Employee or intermediate							0	
occupation							-2.1 (12.7)	
Managerial or academic								
**Country of birth**		0.29		**0.03**		**<0.01**		0.68
Belgium	0		0		0		0	
EU	17.7 (22.8)		**39.6 (17.1)**		**64.5 (29.0)**		-8.8 (15.7)	
Outside the EU	17.0 (12.3)		30.5 (23.2)		**-25.5 (10.7)**		-13.8 (19.5)	
**Language spoken at home**		**<0.01**		0.36		0.66		0.07
French and/or Dutch	0		0		0		0	
Mixed including French or Dutch	-4.1 (8.7)		8.8 (14.5)		15.5 (21.7)		**-24.7 (10.9)**	
Language other than French or	**-26.9 (8.6)**		26.8 (20.1)		-11.4 (21.6)		12.5 (33.2)	
Dutch								
**Region of residency**		**<0.001**		**<0.001**		**<0.001**		**<0.001**
Flanders	0		0		0		0	
Brussels	**-18.7 (8.1)**		**-23.6 (8.7)**		**-27.0 (13.1)**		-22.6 (15.9)	
Wallonia	**-35.4 (5.4)**		**-32.4 (7.1)**		**-39.1 (8.9)**		**-39.5 (9.3)**	
**Refined starchy food, potatoes and tubers**								
**Gender**		**<0.01**		**<0.001**		0.10		0.07
Male	**26.7 (9.7)**		**74.7 (11.5)**		29.8 (18.0)		26.4 (14.7)	
Female	0		0		0		0	
**Household type**		0.12		0.27				
Two-parent family	0		0					
Single-parent family	14.9 (9.6)		13.2 (11.9)		-		-	
Single					**-100.8 (42.7)**	**0.01**	7.6 (28.6)	0.90
Single-parent family					-19.4 (19.0)		26.1 (26.4)	
Couple without children	-		-		**-70.7 (23.5)**		0	
Two-parent family					0		10.5 (18.4)	
Other					-9.2 (22.5)		18.6 (37.6)	
**Highest education level in the**		0.37		0.53		0.79		
**household**	15.2 (10.8)		1.5 (12.1)		-3.3 (19.0)			

Secondary education or lower	8.8 (10.2)		-11.5 (13.9)		-15.2 (23.6)		-	
Bachelor's degree or equivalent	0		0		0			
Postgraduate education								
**Education level of the responder**								0.38
Secondary education or lower							22.3 (16.5)	
Bachelor's degree or equivalent	-		-		-		5.6 (16.3)	
Postgraduate education							0	
**Maternal occupation**		0.07		0.18				
Inactive	**62.0 (22.8)**		-33.8 (17.9)					
Manual worker	**65.3 (24.6)**		**-50.7 (21.0)**					
Self-employed	0		0		-		-	
Employee or intermediate	43.7 (20.9)		**-42.2 (16.6)**					
occupation	41.0 (24.8)		-32.5 (27.3)					
Managerial or academic	33.9 (26.6)		-34.5 (23.1)					
Not applicable (no mother declared)								
**Working status of the responder**						0.06		
Student					**42.7 (17.9)**			
Inactive	-		-		28.1 (22.5)		-	
Active					0			
**Occupation of the responder**								0.13
Inactive							12.3 (16.7)	
Manual worker							34.8 (20.9)	
Self-employed	-		-		-		**69.4 (31.7)**	
Employee or intermediate							0	
occupation							17.1 (19.6)	
Managerial or academic								
**Country of birth**		0.55		0.94		0.99		0.70
Belgium	0		0		0		0	
EU	-3.9 (24.2)		-4.9 (22.3)		-2.5 (36.2)		-7.9 (24.4)	
Outside the EU	-31.8 (29.2)		-6.5 (22.7)		5.1 (33.3)		-21.1 (26.2)	
**Language spoken at home**		0.08		**0.04**		0.07		0.34
French and/or Dutch	0		0		0		0	
Mixed including French or Dutch	14.5 (15.3)		14.4 (19.2)		**55.9 (24.5)**		-10.0 (24.6)	
Language other than French or	**60.2 (28.6)**		**-46.5 (20.2)**		-9.5 (45.3)		-57.7 (40.4)	
Dutch								
**Region of residency**		**<0.001**		**<0.01**		0.77		0.06
Flanders	0		0		0		0	
Brussels	**34.9 (16.1)**		38.0 (23.9)		7.5 (20.2)		2.8 (27.5)	
Wallonia	**35.6 (8.7)**		**32.9 (10.9)**		12.2 (16.9)		**34.2 (14.3)**	

Sugary sweetened beverages

**Gender**		0.83		0.69		0.08		0.53
Male	6.6 (29.8)		14.0 (35.0)		99.6 (56.3)		26.6 (41.8)	
Female	0		0		0		0	
**Household type**		0.17		0.56				
Two-parent family	0		0					

Single-parent family	51.8 (37.7)		22.7 (38.9)		-		-	
Single					159.4 (170.5)	0.26	22.3 (64.0)	0.96
Single-parent family					145.5 (88.4)		0	
Couple without children	-		-		3.3 (86.6)		46.8 (69.7)	
Two-parent family					0		32.1 (58.0)	
Other					131.2 (102.5)		47.8 (83.7)	
**Highest education level in the**		**<0.001**		**<0.001**		0.14		
**household**	**169.6 (33.2)**		**149.9 (37.0)**		125.4 (81.2)			
Secondary education or lower	**128.3 (35.8)**		**128.0 (44.8)**		-11.4 (75.6)		-	
Bachelor's degree or equivalent	0		0		0			
Postgraduate education								
**Education level of the responder**								**<0.001**
Secondary education or lower							**215.9 (51.4)**	
Bachelor's degree or equivalent	-		-		-		39.3 (29.2)	
Postgraduate education							0	
**Maternal occupation**		**0.01**		0.10				
Inactive	**135.4 (57.6)**		**165.2 (82.6)**					
Manual worker	**240.8 (70.9)**		**239.4 (102.3)**					
Self-employed	49.5 (70.1)		117.7 (94.9)		-		-	
Employee or intermediate	**140.1 (57.2)**		86.2 (79.0)					
occupation	0		0					
Managerial or academic	142.2 (98.8)		138.5 (90.0)					
Not applicable (no mother declared)								
**Working status of the responder**						0.13		
Student					0			
Inactive	-		-		221.0 (137.3)		-	
Active					89.3 (59.3)			
**Occupation of the responder**								**<0.001**
Inactive							**213.0 (84.2)**	
Manual worker							**212.8 (68.8)**	
Self-employed	-		-		-		90.9 (79.1)	
Employee or intermediate							7.2 (40.8)	
occupation							0	
Managerial or academic								
**Country of birth**		0.46		0.14		**0.01**		0.39
Belgium	0		0		0		0	
EU	-66.7 (54.4)		-89.9 (69.7)		35.7 (177.5)		-10.9 (56.8)	
Outside the EU	13.0 (79.3)		-91.3 (56.8)		**-163.1 (56.5)**		139.2 (110.3)	
**Language spoken at home**		0.26		0.44		0.12		0.49
French and/or Dutch	0		0		0		0	
Mixed including French or Dutch	-52.1 (36.0)		55.8 (45.7)		113.7 (112.3)		68.6 (62.7)	
Language other than French or	-47.4 (53.9)		-14.8 (64.4)		-140.5 (82.7)		-29.7 (76.4)	
Dutch								
**Region of residency**		0.06		0.37		**0.001**		0.32

Flanders	0	0	0	0
Brussels	-76.3 (39.6)	-68.8 (50.3)	**-165.1 (50.4)**	-63.4 (49.6)
Wallonia	26.9 (34.2)	-28.5 (37.5)	-13.3 (69.8)	23.2 (47.99)

[a] Adjusted for total energy intake; **bold**: category for which consumption statistically significantly differed from reference category (p<0.05); -: variable not concerning the age group.

Table S2. Description of the study sample. Adolescents and young adults, Belgian Food Consumption Survey 2014.

	Age category							
	10-13 years *n*= 447		14-17 years *n*= 470		18-25 years *n*= 233		26-39 years *n*= 355	
	n	%[a]	n	%[a]	n	%[a]	n	%[a]
**Gender**								
Male	209	51.3	237	51.1	114	49.7	188	52.3
Female	238	48.7	233	48.9	119	50.3	167	47.7
**Household type**								
Two-parent family	328	74.6	325	69.6	-	-	-	-
Single-parent family	113	25.4	141	30.4				
Single					15	7.0	39	11.9
Single-parent family					50	24.0	29	8.0
Couple without children	-	-	-	-	16	6.0	68	19.0
Two-parent family					128	54.5	191	53.8
Other					24	8.5	28	7.3
**Highest education level in the household**								
Secondary education or lower	149	34.6	162	33.2	78	34.5		
Bachelor's degree or equivalent	135	31.3	139	30.4	89	36.2	-	-
Postgraduate education	153	34.1	162	36.4	62	29.3		
**Education level of the responder**								
Secondary education or lower							120	32.3
Bachelor's degree or equivalent	-	-	-	-	-	-	105	32.2
Postgraduate education							127	35.5
**Maternal occupation**								
Inactive	81	18.6	83	17.1				
Manual worker	49	9.7	49	10.4				
Self-employed	28	7.2	38	9.1	-	-	-	-
Employee or intermediate occupation	231	51.6	247	53.1				
Managerial or academic	28	5.4	18	3.3				
Not applicable (no mother declared)	30	7.5	35	7.0				
**Working status of the responder**								
Student					139	60.9		
Inactive	-	-	-	-	20	7.2	-	-
Active					74	31.9		

**Occupation of the responder**								
Inactive							57	14.7
Manual worker							62	17.0
Self-employed	-	-	-	-	-	-	25	6.9
Employee or intermediate occupation							177	52.0
Managerial or academic							34	9.4
**Country of birth**								
Belgium	407	91.6	434	91.8	212	87.5	317	89.7
EU	23	4.8	20	4.6	9	6.5	14	3.8
Outside the EU	17	3.6	16	3.6	12	6.0	24	6.5
**Language spoken at home**								
French and/or Dutch	393	86.6	418	86.8	216	92.0	323	91.3
Mixed including French or Dutch	38	10.2	38	10.1	8	4.4	19	5.4
Language other than French or Dutch	16	3.2	14	3.1	9	3.6	13	3.3
**Region of residency**								
Flanders	247	56.4	268	57.4	138	59.8	209	57.8
Brussels	37	10.8	40	9.7	14	11.2	30	11.8
Wallonia	163	32.8	162	32.9	81	29.0	116	30.4

[a] weighted proportions; -: variable not concerning the age group.
